# Intravascular Optical Coherence Tomography for Characterization of Atherosclerosis with a 1.7 Micron Swept-Source Laser

**DOI:** 10.1038/s41598-017-15326-4

**Published:** 2017-11-06

**Authors:** Yan Li, Joseph Jing, Emon Heidari, Jiang Zhu, Yueqiao Qu, Zhongping Chen

**Affiliations:** 1 Beckman Laser Institute, University of California, Irvine, 1002 Health Sciences Road, Irvine, CA 92617 USA; 2 Department of Biomedical Engineering, University of California Irvine, Irvine, CA 92697-2700 USA

## Abstract

The main cause of acute coronary events, such as thrombosis, is the rupture of atherosclerotic plaques. Typical intravascular optical coherence tomography (IVOCT) imaging systems that utilize a 1.3 μm swept source laser are often used for identifying fibrous cap thickness of plaques, yet cannot provide adequate depth penetration to resolve the size of the lipid pool. Here, we present a novel  IVOCT system with a 1.7 μm center wavelength swept light source that can readily penetrate deeper into the tissue because of the longer wavelength and allows for better identification of plaques due to the lipid absorption spectrum at 1.7 μm. Using this system, we have imaged a human coronary artery to evaluate the performance of the novel OCT system and verified the results by hematoxylin and eosin (H&E) histology. The significantly improved imaging depth and better identification sensitivity suggest that the 1.7 μm OCT system holds great potential  that can be further translated for *in-vivo* applications of atherosclerosis characterization.

## Introduction

Coronary artery disease is the most common type of heart disease in developed countries with high mortality caused by ruptured atherosclerotic plaques. Accurate assessment of vulnerable atherosclerotic plaques is essential for choosing proper interventional techniques. According to prior clinical studies, there are three characteristics of vulnerable plaques that are used as the criteria to estimate whether the plaque is vulnerable: (i) a large lipid pool, (ii) thin fibrous cap, and (iii) major inflammatory reaction^1–4^. Various imaging technologies, such as intravascular optical coherence tomography (IVOCT), intravascular ultrasound (IVUS), and a combined multimodality imaging system, have been developed to detect vulnerable plaques with the hope of guiding therapy and monitoring response to intervention^5–10^. IVOCT, the latest development in intravascular coronary imaging, offers cross-sectional images of human arteries with a superior spatial resolution of ~15 μm that enables the detection of micrometer-scale features of atherosclerosis such as the intimal cap layers associated with vulnerable plaques^11–14^.

In the clinic, the IVOCT system based on a swept source laser with a center wavelength of 1.3 μm is often used for identifying the thin fibrous cap^6^. However, limited by the depth penetration of the typical IVOCT system, it is difficult to visualize the large lipid pool. Therefore, IVUS with an imaging depth of ~7 mm and a resolution of ~150 μm is utilized to image both the lumen geometry and structure of the arterial wall that may contain large lipid pools^10,15,16^. The IVOCT system with a center wavelength at 1.7 μm has several advantages compared to the IVOCT system at 1.3 μm. First, the contrast between lipid and normal tissue will be more obvious, based on stronger lipid absorption^17^. In addition, the light at a wavelength of 1.7 μm can penetrate deeper into tissue; thus, more structural information can be obtained, enabling the visualization of a large lipid pool. Here, we present a novel IVOCT system with a 1.7 μm swept source laser for identification of atherosclerosis. We performed imaging in phantom and human coronary artery specimens. Our results indicate that the IVOCT system with a center wavelength of 1.7 μm increases the imaging depth and allows for better identification of the morphology and chemical composition of atherosclerotic plaques.

## Results

### Phantom preparation and imaging

**Figure 1:**
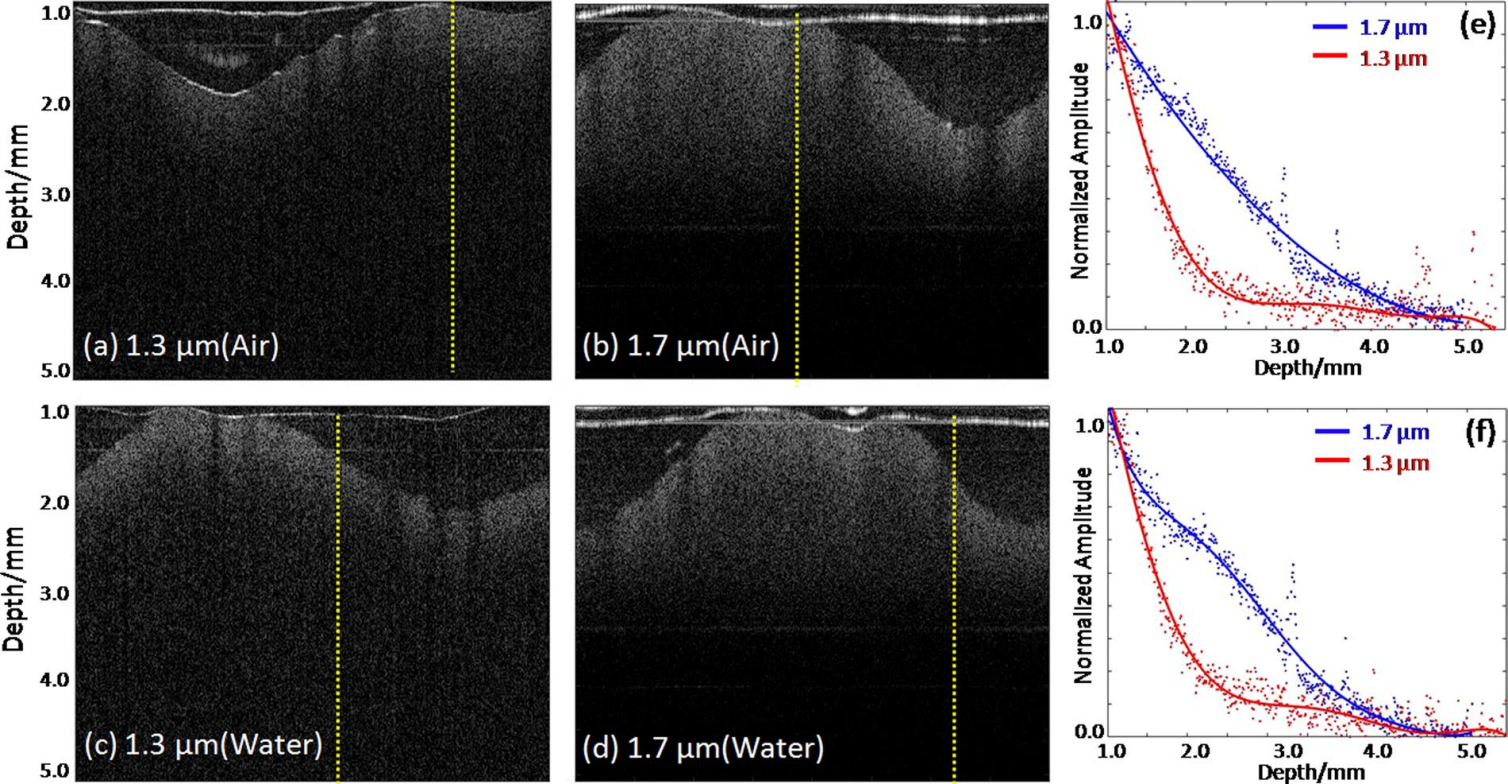
IVOCT images of vessel-mimicking phantom and quantitative analysis for penetration depth. (**a**) and (**c**) IVOCT images with conventional IVOCT system in air and water, respectively. (**b**) and (**d**) IVOCT images with the 1.7 μm IVOCT system in air and water, respectively. (**e**) and (**f**) Quantitative analysis of penetration depth for two IVOCT systems in air and water.

In order to demonstrate the performance of the novel IVOCT system, a vessel-mimicking gelatin phantom was imaged. A solution containing 5% gelatin and 2% silica particles with an average particle diameter of 5 μm was molded into a hollow cylindrical shape. Figure 1(a–d) show IVOCT images of the phantom with the novel (1.7 μm) and conventional (1.3 μm) IVOCT systems. Figure 1(a and b) show the phantom images in air. Figure 1(c and d) demonstrate the phantom images in water. Between Fig. 1(a and b), the penetration depth is deeper for the 1.7 μm OCT system compared to the 1.3 μm OCT system. Figure 1(e) is a quantitative analysis of the imaging depth that shows the relationship between the depth and the OCT signal intensity from the area indicated by the yellow dashed line in Fig. 1(a and b). From Fig. 1(e), we can conclude that the penetration depth of the 1.7 μm IVOCT system is approximately two times higher than that of the 1.3 μm IVOCT system. In consideration of the strong absorption from water at the 1.7 μm wavelength, the experiments were also performed in water for a more accurate estimation. The corresponding IVOCT images are shown in Fig. 1(c and d). Figure 1(f) is a quantitative analysis from the area indicated by yellow dashed line in Fig. 1(c and d), which also shows an increase in penetration depth for the 1.7 μm OCT system compared to the 1.3 μm OCT system. By comparing the penetration depth results in both water and air, we can infer that the absorption from water does not influence the penetration depth significantly, with the reason being that the volume of the phantom’s hollow cylinder is relatively small. With regards to an *in-vivo* clinical application, the lumen’s coronary artery diameter is about 2–3 mm, which is small enough such that the influence from the water absorption is negligible.

### Human Coronary Artery

**Figure 2:**
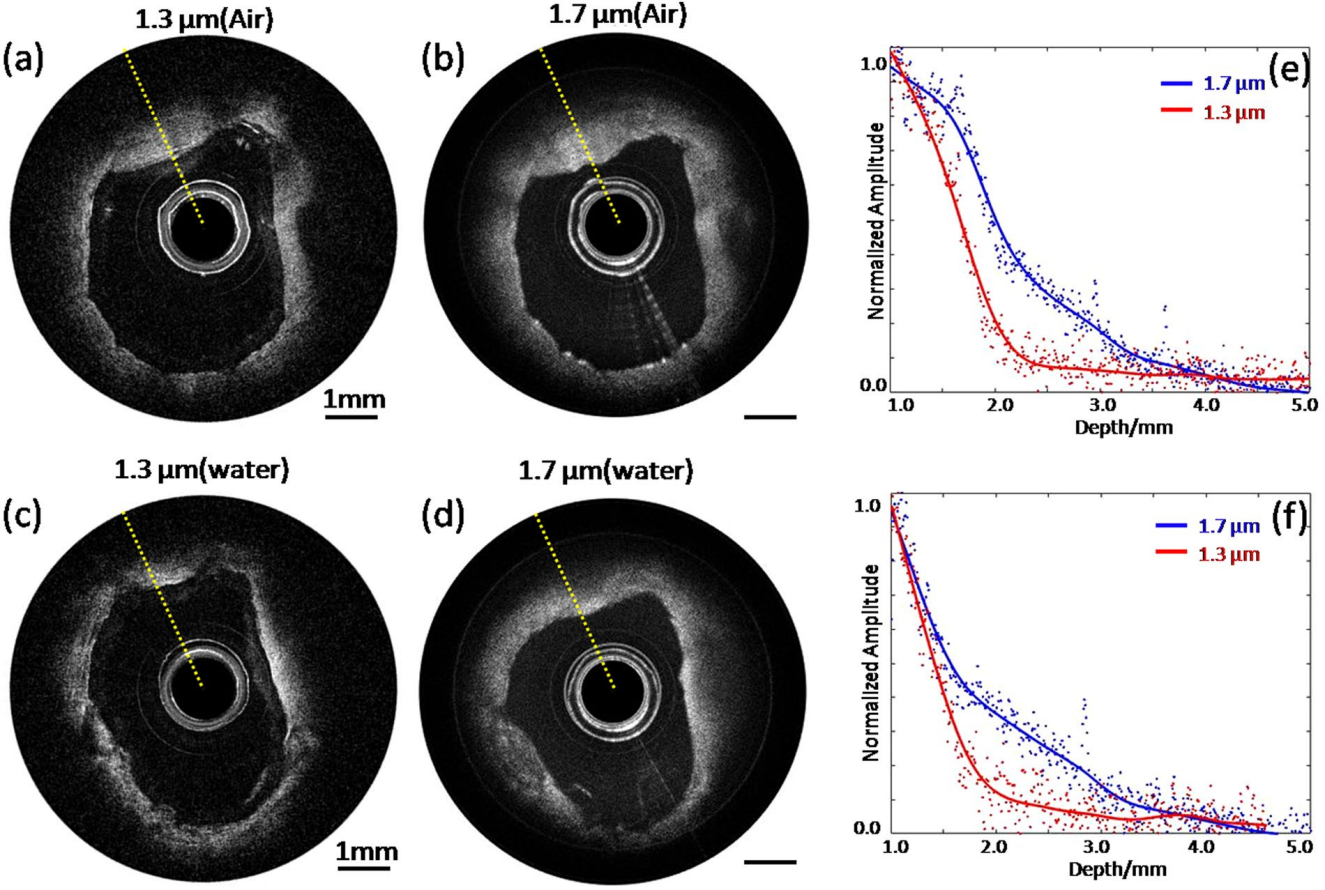
IVOCT images of healthy human artery and quantitative analysis for penetration depth. (**a**) and (**c**) IVOCT images with the 1.3 μm IVOCT system in air and water, respectively. (**b**) and (**d**) IVOCT images with the 1.7 μm IVOCT system in air and water, respectively. (**e**) and (**f**) Quantitative analysis of penetration depth for two IVOCT systems in air and water. Scale bars are 1 mm.

To further verify the capability of large penetration depth with the 1.7 μm IVOCT system, a healthy human coronary artery was imaged. Figure 2(a and c) show IVOCT images obtained by the 1.3 μm IVOCT system in air and water, respectively. Figure 2(b and d) show IVOCT images obtained by the 1.7 μm IVOCT system in water and air, respectively. Comparing Fig. 2(a,b,c and d), it can be seen that more information was obtained along the axial direction with the 1.7 μm IVOCT system. Figure 2(e and f) are the quantitative analyses of penetration depth for the two IVOCT systems in air and water, respectively, which also demonstrate the larger penetration depth achievable with the 1.7 μm IVOCT system. These results agree well with the phantom experiments.

**Figure 3:**
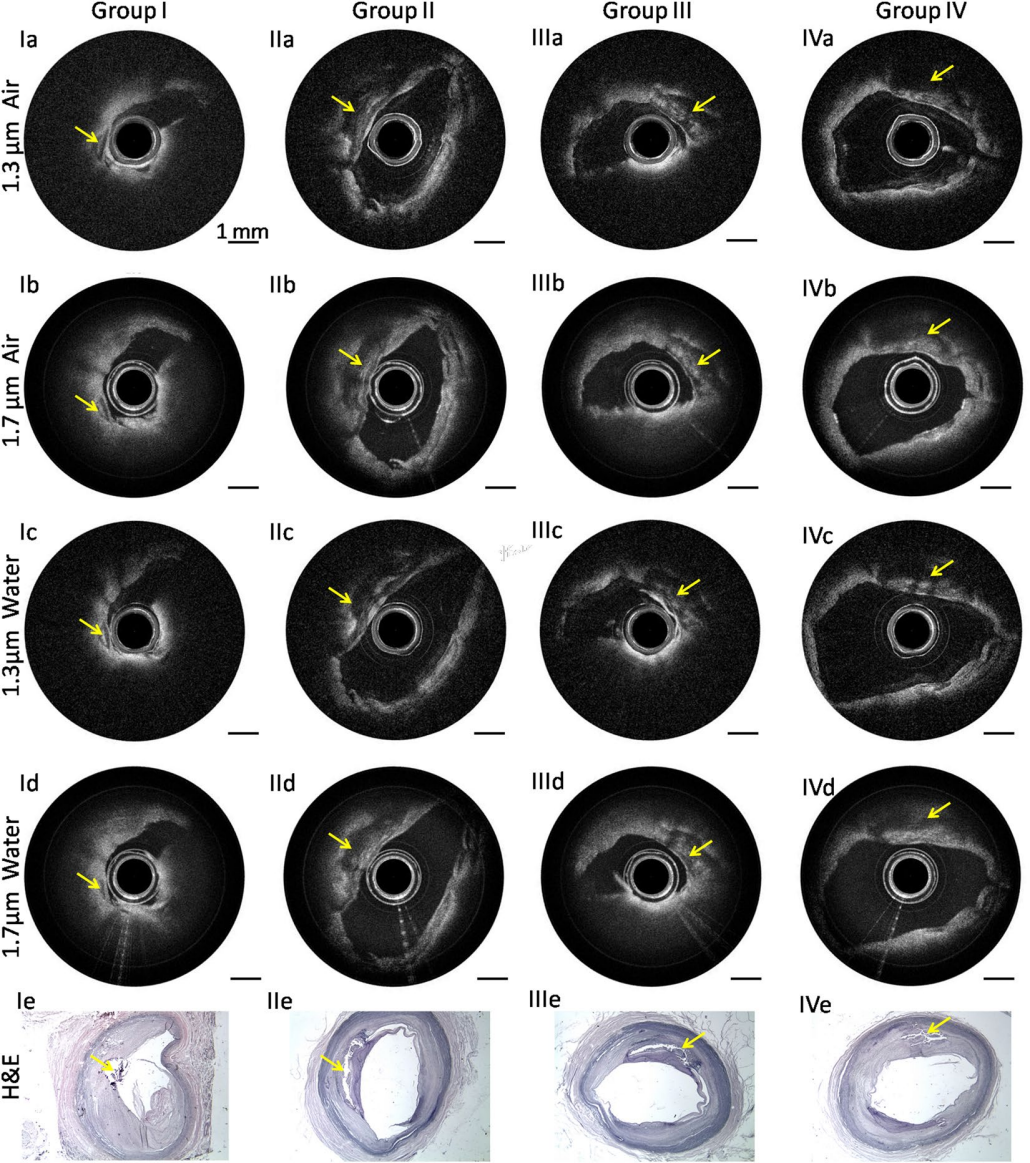
IVOCT images of the atherosclerotic coronary artery. (**Ia**–**IVa**) IVOCT images obtained by the 1.3 μm IVOCT system in air. (**Ib**–**IVb**) IVOCT images obtained by the 1.7 μm IVOCT system in air. (**Ic**–**IVc**) IVOCT images obtained by the 1.3 μm IVOCT system in water. (Id-IVd) IVOCT images obtained by the 1.7 μm IVOCT system in water. (**Ie**–**IVe**) Hematoxylin and eosin (H&E) histology. Scale bars are 1 mm.

To demonstrate the capability of differentiating the plaque from normal tissue, atherosclerotic coronary arteries were imaged by using the two IVOCT systems. Groups (I-IV) are IVOCT images at different arterial sites with different pathological features. Figure 3 (a–d) were obtained at similar sites by the 1.3 μm and 1.7 μm IVOCT systems in both air and water. Figure 3 (Ie–IVe) are corresponding hematoxylin and eosin (H&E) histology for each group. For group I, a low-density signal region (denoted by the yellow arrow) can be found in Fig. 3 (Ia–Ie), which indicates the existence of a calcified plaque. The classification of the plaque type is validated by the corresponding histology images, which match the four IVOCT images well. For groups II and IV, a large low-density signal region was also found, which indicates the existence of thick-cap (>65 μm) fibroatheroma (ThCFA). The corresponding H&E histology [Fig. 3 (II e and IVe)] all verified the results. For group III (a-d), a thin fibrous cap and a large low-density signal region behind the thin fibrous cap were found, indicating thin-cap (<65 μm) fibroatheroma (TCFA). Figure 3 (IIIe) shows the corresponding histology, which verified these results. From the corresponding images of the two systems, it is clearly seen that the 1.7 μm IVOCT system images have a larger penetration depth compared to the 1.3 μm IVOCT system images, which demonstrates the capability of the 1.7 μm system to visualize the whole plaque. Analyzing the results in water and air, we can find that the absorption of water is almost negligible for the atherosclerotic coronary artery due to the small lumen diameter. These IVOCT and H&E histology images illustrate the capability of the 1.7 μm system to identify the plaque with a large imaging depth and high sensitivity.

## Discussion

IVOCT, a minimally invasive, non-ionizing imaging technology, is the gold standard to detect the thin fibrous cap of vulnerable plaques. Due to the shallow penetration depth of the 1.3 μm IVOCT systems, the capability of identifying a large lipid pool is often limited. Here, we utilized a 1.7 μm swept source laser for the IVOCT system that realized a higher sensitivity based on the lipid absorption spectrum and an improved depth penetration based on a longer wavelength band. The results from the human coronary artery *in-vitro* experiments with the corresponding histopathology verified the performance of the 1.7 μm IVOCT system.

Although our results clearly demonstrate the advantages of the 1.7 μm IVOCT system, the current 1.7 μm IVOCT system is far from optimal. The reason is that our current optical couplers work at a center wavelength of either 1.3 μm or 1.5 μm, which reduces the output energy of the light source. In addition, the detector used in the 1.7 μm IVOCT system has a wavelength sensitivity range of 800–1700 nm and is optimized at 1310 nm, which decreases the detection efficiency significantly. If custom-made couplers and detectors that are optimized for the 1.7 μm wavelength are implemented, the performance of the 1.7 μm IVOCT system will be significantly improved.

In summary, the novel IVOCT system with the 1.7 μm swept source laser provides a new insight into the pathology of coronary artery disease *in-vivo* and is a powerful tool to assess the immediate and long-term outcomes of percutaneous coronary intervention. Moreover, by combining with other imaging modalities, such as ultrasound imaging and elastography^18,19^, it will provide the physician with a powerful tool for imaging, diagnosing, and managing vulnerable plaques. Furthermore, the spectroscopic IVOCT system can also be developed to increase contrast for lipid identification.

## Methods

### High speed scanning laser

**Figure 4:**
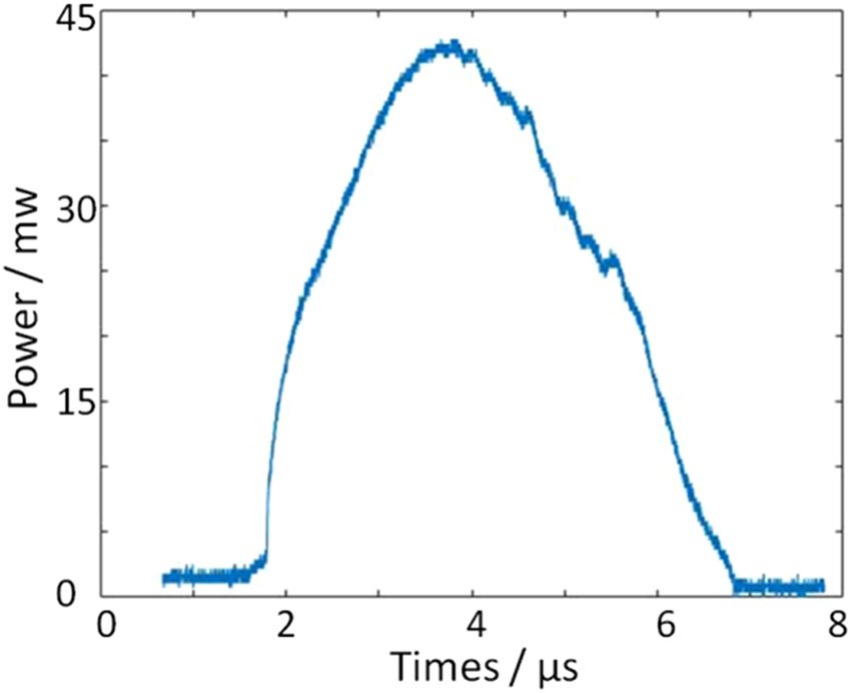
The output power of the 1.7 μm swept source laser.

We have collaborated with Santec, Inc., to develop a novel 1.7 μm IVOCT system. The high speed scanning laser is a key component. Table 1 shows the parameters of this laser, and Fig. 4 shows the output power over time. For characterization of atherosclerosis, this laser has several advantages. First, its tuning range is from 1600 nm to 1770 nm, which covers the absorption peak of lipid, the main content of vulnerable plaques. Therefore, the 1.7 μm IVOCT system will provide a high sensitivity for lipid content. In addition, the long wavelength range and high output power will contribute to a large penetration depth and better signal-to-noise ratio which makes the visualization of the whole plaque possible. All of these features make the 1.7 μm IVOCT system a better tool for the characterization of atherosclerosis.

**Table 1:** Parameters of the high speed scanning laser.

Parameter	Units	Min	Max	Measured
Maximum Optical Output Power	mW	35	—	42.8
Scan Range	nm	135	—	173.8
Center Wavelength	nm	1665	1725	1684.9
Coherence Length	mm	8	—	10.0
Scan Rate	kHz	89.9	90.1	90.0

### System setup and imaging probe

**Figure 5:**
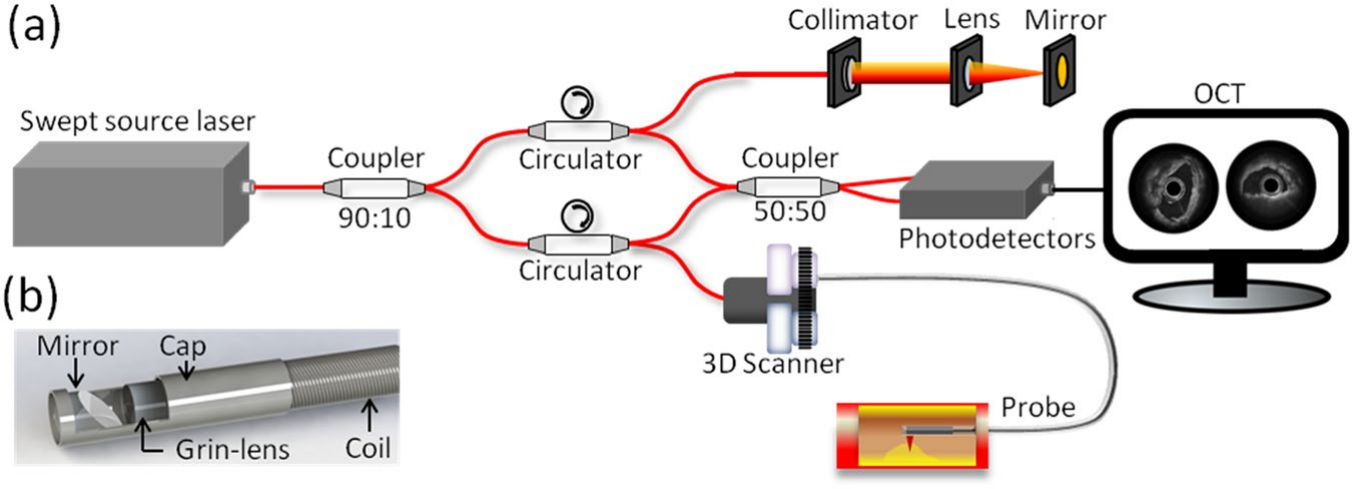
Schematic of the 1.7 μm IVOCT system (**a**) and the imaging probe (**b**).

The novel IVOCT system was built based on a 1.7 μm swept source laser with a sweep rate of 90 kHz as shown in Fig. 5. Because there is no commercial coupler and detector specifically designed for the 1.7 μm wavelength band, we applied the couplers (90:10 and 50:50) with a center wavelength of 1310 nm and circulators with a center wavelength of 1550 nm in the proposed IVOCT system. The mismatch between the couplers, circulars, and swept source laser caused a decrease in  the laser power. In order to demonstrate the performance of the novel IVOCT, we also built a typical 1.3 μm IVOCT system with the same optical components except for the circulators. A swept source (Santec, Inc., HSL-2100) with a center wavelength of 1310 nm and a sweeping rate of 20 kHz was used in the 1.3 μm OCT system. In both IVOCT systems, output light was split by a 90:10 coupler into the sample and reference arms, respectively. A balanced photodetector (800 nm-1700 nm) and a 12 bit data acquisition board were used to detect and record the interference signal. The IVOCT software was written entirely in C++ for data acquisition, image processing, and display in real-time using GPU.

A 1.2 mm proximal scanning endoscopic OCT probe^20^ was developed for 3-D imaging as shown in Fig. 5. The OCT laser beam propagates through the single mode core fiber, is focused by a grin-lens, and then reflected to the tissue surface by a rod mirror at an angle of 45°. The spot size at a 1.5 mm working distance (1700 nm and 1310 nm) is around 15 µm. All of the elements were housed in a metal cap and fixed by epoxy. The cap was connected to a double-wrapped torque coil. A custom-made rotary joint was used to propagate the laser beam while rotating the probe. Two motors were used for driving the rotating/pullback catheter assembly. The pullback speed was set to be 1 mm per second with a frame rate of 40 images (the 1.7 μm OCT system) and 20 images (the 1.3 μm OCT system) per second.

For allexperiments, the output power of the 1.3 μm OCT system was attenuated to the same energy with the 1.7 μm OCT system. The energy from the probe tip was measured to be around 6 mW for both IVOCT systems. Under the same conditions, we performed the experiments and analyzed the results. The two OCT systems have similar sensitivity (1.3 µm: 101 dB, 1.7 µm: 102 dB) and resolution (1.3 µm: 17.9 µm, 1.7 µm: 22.6 µm). In addition, a logarithmic transform was applied to the OCT signal in all of the figures and plots to make low-reflective layers visible.

### Human coronary artery imaging

Fresh human coronary  artery samples were obtained from cadavers and frozen in a −19 degree freezer. After the imaging system was set up, the tissue was imaged with the 1.7 μm IVOCT system and 1.3 μm IVOCT system in air and water. After imaging, the region of interest was marked with pins. The tissue was decalcified, embedded, and sectioned to 6 μm-thick slides. Then the slides were stained with H&E, and images were taken with a microscope with 4x magnification to find a match with the experimental region of interest. All methods were carried out in accordance with the University of California, Irvine (UCI) Institutional Review Board (IRB) and the Institutional Biosafety Committee (IBC). IRB granted an exemption to the protocol requirement since the activities do not constitute Human Subject Research. Informed consent was deemed unnecessary because confidentiality of the deceased cadaver tissues is protected and coded. All experimental protocols were approved by the UCI IBC under protocol #2016–1570.
